# Failure to Furnish Medical Aid—Religious Belief

**Published:** 1904-10

**Authors:** 


### Abstracts and Selections.

### Failure to Furnish Medical Aid—Religious Belief.

The Indiana Supreme Court has held that the father or guardian of a child is bound to furnish that child with medical attention and that religious doctrine or belief can not be accepted as a justification or excuse for failure to do so. (State vs. Chenoweth, 71 N. E. Rep., 198, Ind.) The defendant in this case had ample means to hire medical attention for his child, who was sick. The child had a cough which defendant took to be whooping cough.

The child died of broncho-pneumonia. Defendant stated that he employed "divine healing," that he had an elder come to the house and anoint the child with oil and pray for recovery, and that he had also communicated with Dowie and asked for absent treatment. The court found him guilty of criminal neglect.—*Exchange.*

